# Combatting the effect of image reconstruction settings on lymphoma [^18^F]FDG PET metabolic tumor volume assessment using various segmentation methods

**DOI:** 10.1186/s13550-022-00916-9

**Published:** 2022-07-29

**Authors:** Maria C. Ferrández, Jakoba J. Eertink, Sandeep S. V. Golla, Sanne E. Wiegers, Gerben J. C. Zwezerijnen, Simone Pieplenbosch, Josée M. Zijlstra, Ronald Boellaard

**Affiliations:** 1 Cancer Center Amsterdam, Department of Radiology and Nuclear Medicine, Amsterdam UMC, Vrije Universiteit Amsterdam, De Boelelaan 1117, Amsterdam, The Netherlands; 2 Cancer Center Amsterdam, Department of Hematology, Amsterdam UMC, Vrije Universiteit Amsterdam, De Boelelaan 1117, Amsterdam, The Netherlands

**Keywords:** Lymphoma, [^18^F]FDG PET, Metabolic tumor volume, Reconstruction, Segmentation

## Abstract

**Background:**

[^18^F]FDG PET-based metabolic tumor volume (MTV) is a promising prognostic marker for lymphoma patients. The aim of this study is to assess the sensitivity of several MTV segmentation methods to variations in image reconstruction methods and the ability of ComBat to improve MTV reproducibility.

**Methods:**

Fifty-six lesions were segmented from baseline [^18^F]FDG PET scans of 19 lymphoma patients. For each scan, EARL1 and EARL2 standards and locally clinically preferred reconstruction protocols were applied. Lesions were delineated using 9 semiautomatic segmentation methods: fixed threshold based on standardized uptake value (SUV), (SUV = 4, SUV = 2.5), relative threshold (41% of SUVmax [41M], 50% of SUVpeak [A50P]), majority vote-based methods that select voxels detected by at least 2 (MV2) and 3 (MV3) out of the latter 4 methods, Nestle thresholding, and methods that identify the optimal method based on SUVmax (L2A, L2B). MTVs from EARL2 and locally clinically preferred reconstructions were compared to those from EARL1. Finally, different versions of ComBat were explored to harmonize the data.

**Results:**

MTVs from the SUV4.0 method were least sensitive to the use of different reconstructions (MTV ratio: median = 1.01, interquartile range = [0.96–1.10]). After ComBat harmonization, an improved agreement of MTVs among different reconstructions was found for most segmentation methods. The regular implementation of ComBat (‘Regular ComBat’) using non-transformed distributions resulted in less accurate and precise MTV alignments than a version using log-transformed datasets (‘Log-transformed ComBat’).

**Conclusion:**

MTV depends on both segmentation method and reconstruction methods. ComBat reduces reconstruction dependent MTV variability, especially when log-transformation is used to account for the non-normal distribution of MTVs.

**Supplementary Information:**

The online version contains supplementary material available at 10.1186/s13550-022-00916-9.

## Background

Positron emission tomography (PET) and computed tomography (CT) are oncological imaging modalities extensively used for staging and treatment response assessment in lymphoma [1]. Alone and when combined with existing prognostic indicators, quantitative imaging characteristics extracted from PET scans have been shown to improve risk stratification. Baseline metabolic tumor volume (MTV) is a quantitative measure, obtained from [^18^F]-fluorodeoxyglucose ([^18^F]FDG) PET scans, which quantifies tumor burden with FDG uptake [2]. Several studies demonstrated that high MTV before starting treatment is significantly correlated with a shorter progression-free survival (PFS) and/or overall survival (OS) [3, 4]. These findings imply that MTV is a promising prognostic factor in tailoring lymphoma therapy. However, quantitative PET measures are susceptible to image quality in varying degrees, including the different PET reconstruction methods [5–7]. As such, several papers clearly demonstrated the high sensitivity of intensity measures like the maximal standardized uptake value (SUVmax), SUVpeak and multiple textural features for reconstruction setting [11, 14, 15]. As a result, new image reconstruction methods, such as the point spread function (PSF), pose uncertainties about their impact on the different quantitative PET metrics, including volumetric features like MTV [8, 9]. Despite the auspicious potential clinical value of MTV as a prognostic and response predictive marker in lymphoma, susceptibility to reconstruction setting and thus inability to reliably reflect (changes in) tumor burden precludes any clinical implementation.

In this light, we aim to evaluate whether, and to which extent, MTV is sensitive to reconstruction setting including the impact of segmentation method. Therefore, in this study we assess the variability in MTV using 9 semiautomatic segmentation methods to variations in 3 different image reconstruction methods. Throughout the literature, the ComBat method has been proposed as a solution to reduce the impact of the image preprocessing effect and it is currently used in various contexts [10]. Originally, ComBat has been used in genomics as a harmonization strategy to deal with the alterations caused by batch effects [11]. When applying this method, the batch effect is discarded as all data are realigned in a single space and biological information remains unchanged. In image analysis, we can use ComBat to compensate for the variability among features generated by the scanner/protocol effect. However, ComBat harmonization is not always correctly used. Therefore, in this paper we also analyze the ability of using ComBat to remove variability in MTV and whether different implementations of ComBat are able to further improve the alignment of the data.

## Methods

### Study population

In this study, we used baseline [^18^F]FDG PET/CT scans from 19 patients from two different datasets. The first dataset consists of 14 patients scanned at Amsterdam UMC which were retrospectively obtained from ongoing studies with a waiver for informed consent from the Medical Ethics Review Committee of Amsterdam UMC, location VUmc (IRB2018.029). From these 14 patients, 9 patients were diagnosed with DLBCL, 3 were diagnosed with Hodgkin lymphoma, 2 were diagnosed with T cell lymphoma and 1was diagnosed with post-transplant lymphoproliferative disorder (PTLD). The second dataset consists of 5 DLBCL patients which were recruited at the outpatient clinics of the department of Hematology of the Amsterdam UMC, location VUmc, and the outpatient clinics of the department of Hematology of the Amstelland Hospital in Amstelveen (IRB2019.278). These trials enrolled patients aged 18 years or older diagnosed with DLBCL with at least one tumor with a diameter equal to or more than 3 cm. Patients who had undergone chemotherapy in the past 4 weeks showed multiple malignancies, metal implants or pregnant/lactating patients were excluded from the study.

### Quality control of scans

The quality control (QC) check of the scans followed the EANM guidelines: The liver SUVmean should be between 1.3 and 3.0, and the plasma glucose should be lower than 11 mmol/L [12]. Furthermore, scans were excluded during the QC if the scans were incomplete and/or the total image activity (MBq) was not between 50 and 80% of the total injected FDG activity and/or any DICOM data were missing as in [2].

### Image processing

In order to analyze the impact of reconstruction methods, we used [^18^F]FDG PET baseline scans derived from three different reconstruction methods: one reconstruction which followed locally clinically preferred protocols (high resolution or HR reconstruction), another reconstruction following EARL1 standards (EARL1 reconstruction) and a third reconstruction following EARL2 standards (EARL2 reconstruction). EARL2 standards were established with the implementation of PSF into the original EARL image reconstruction capabilities [13]. PSF is a resolution modeling algorithm which improves image resolution and contrast [14]. In comparison with EARL standards, the most substantial configuration to the HR reconstruction is a pixel spacing parameter of 2 mm instead of 4 mm and a higher spatial resolution. Table 1 contains a summary of the parameters related to the reconstruction methods  used in this study.

**Table 1:** Summary of parameters characterizing each reconstruction method

Method	Series description	Pixel spacing (mm)	Slice thickness (mm)	Reconstruction method	Manufacturer’s model name
EARL1	[WBA_CTAC]-Body	4 × 4 × 4	4	BLOB-OS-TF	Ingenuity TF PET/CT, Vereos PET/CT
EARL2	[WBA_CTAC_PSF]-Body	4 × 4 × 4	4	BLOB-OS-TF	Ingenuity TF PET/CT, Vereos PET/CT
HR	[HN_CTAC_2mm]-Body	2 × 2 × 2	2	BLOB-OS-TF	Ingenuity TF PET/CT, Vereos PET/CT

The MTV of lesions was calculated and analyzed using ACCURATE software [15]. ACCURATE enables the calculation of MTV of lesions on PET scans automatically and allows the users to apply multiple segmentation methods or volumes of interest (VOI) [15]. Nineteen lymphoma patients were included in the analysis. For each PET baseline study, 3 different reconstructions were investigated (EARL1, EARL2 and HR). We delineated on average 3 lesions per PET scan, which resulted in a total of 56 lesions across all of the included patients. Nine different semiautomatic segmentation methods were applied to delineate each of these lesions. Since each PET scan consisted of 3 reconstructions, a total of 1512 delineations and MTV measurements were included for the analysis.

For each reconstructed scan, the following segmentation methods were applied: segmentation based on fixed thresholds using standardized uptake value of 4.0 (SUV4.0), and SUV of 2.5 (SUV2.5), 41% of SUVmax (41M), segmentation based on adaptive thresholding using 50% of peak voxel value adapted for local background (A50P), majority vote approaches for segmenting voxels detected by at least 2 (MV2) and 3 (MV3) out of these 4 methods [16], lesional-based methods that identify the optimal method based on SUVmax (L2A, L2B) [17] and a contrast oriented method, Nestle segmentation [18]. For the L2A method, a SUV4.0 contour is used for SUVmax > 10 and MV3 for SUVmax < 10. For L2B, MV2 instead of SUV4.0 in case of SUVmax > 10 was used. The majority vote approaches are based upon the agreements between SUV4.0, SUV2.5, A50P and 41M. A detailed description of the methods can be found in [19].

### ComBat harmonization

ComBat harmonization was applied to align the MTV measurements from the three different reconstructions used in this study. As aforementioned, ComBat was first described in the field of genomics to remove batch effects [10, 20]. The ComBat method assumes that the deviation introduced by the batch effect is removed once the means and the variances are standardized across the different batches. The value of the feature *Y* for a specific VOI *j* and scanner *i* is expressed as follows:

1 $$Y_{ij} = \alpha + \gamma_{i} + \delta_{i} \varepsilon_{ij} ,$$

where $\alpha$ represents the mean value of the feature *Y*, $\gamma$ represents the additive effect of the scanner, $\delta$ is the multiplicative effect of the scanner, and $\varepsilon$ is the error. In this case, the feature *Y* would be the MTV and the VOI *j* the delineated lesion. This harmonization method uses the empirical Bayes framework to estimate the batch/scanner effect terms, $\gamma_{i}$ and $\delta_{i}$. Subsequently, the corrected *Y* value $Y_{ij}^{{{\text{ComBat}}}}$ is calculated in Eq. (2) where $\hat{\alpha }$, $\widehat{{\gamma_{i} }}$ and $\widehat{{\delta_{i} }}$ are estimations of parameters $\alpha$, $\gamma_{i}$ and $\delta_{i},$ respectively.

2 $$Y_{ij}^{{{\text{ComBat}}}} = \frac{{Y_{ij} - \hat{\alpha } - \widehat{{\gamma_{i} }}}}{{\widehat{{\delta_{i} }}}} + \hat{\alpha }$$

To understand how the implementation of ComBat is affecting our MTV values, we implemented multiple versions and compared them to the original data. Initially, we applied the regular implementation of ComBat which derives the transformation by aligning the mean and standard deviation of the data groups pertaining to different reconstructions (‘Regular ComBat’). This implementation of ComBat assumes a normal distribution of the data. Since medical data are rarely normally distributed, we also implemented the version of ComBat which applies the logarithmic transformation to attain normal distributions (‘Log-transformed ComBat’). When applying such transformation, the returned values have already been exponentially transformed to be comparable with the rest. Details of these two ComBat versions can be found in Table 2. Another approach to address the non-normal data distribution is to standardize the median and interquartile range instead of the mean and the standard deviation. Furthermore, we investigated whether excluding outliers affects the harmonization of the data. ComBat was applied using R version 4.0.5 based on the code provided by Fortin et al. [21].

**Table 2:** Description of characteristics of ComBat implementations

Version	Alignment	Outliers	Normalization
Regular ComBat	Mean, SD	Include	None
Log-transformed ComBat	Mean, SD	Include	Logarithmic transformation

*SD* Standard Deviation

### Statistical analysis

We first compared the MTV values across the 9 different segmentation methods. For each one of the lesions, we compared the MTVs obtained from EARL2 or HR reconstructions to those from EARL1 using MTV volume ratios. Since EARL1 is used as the reference reconstruction method, in these ratios, EARL1 results are given in the denominator as shown in the following equations:

3 $${\text{MTV}}\;{\text{Ratio}}\;{\text{EARL}}2 = \frac{{{\text{EARL2 }}\;{\text{MTV}}}}{{{\text{EARL}}1 \;{\text{MTV}}}}$$

4 $${\text{MTV}}\;{\text{Ratio }}\;{\text{HR}} = \frac{{{\text{HR}}\;{\text{MTV}}}}{{{\text{EARL1 }}\;{\text{MTV}}}}$$

Equations (3) and (4) were calculated across all of the 9 segmentations which resulted in a MTV ratio value per lesion for each segmentation method for both EARL2 and HR reconstructions. MTV ratios were used to compare the effect of different reconstructions across multiple segmentations before applying ComBat and after applying ComBat.

## Results

**Fig. 1:**
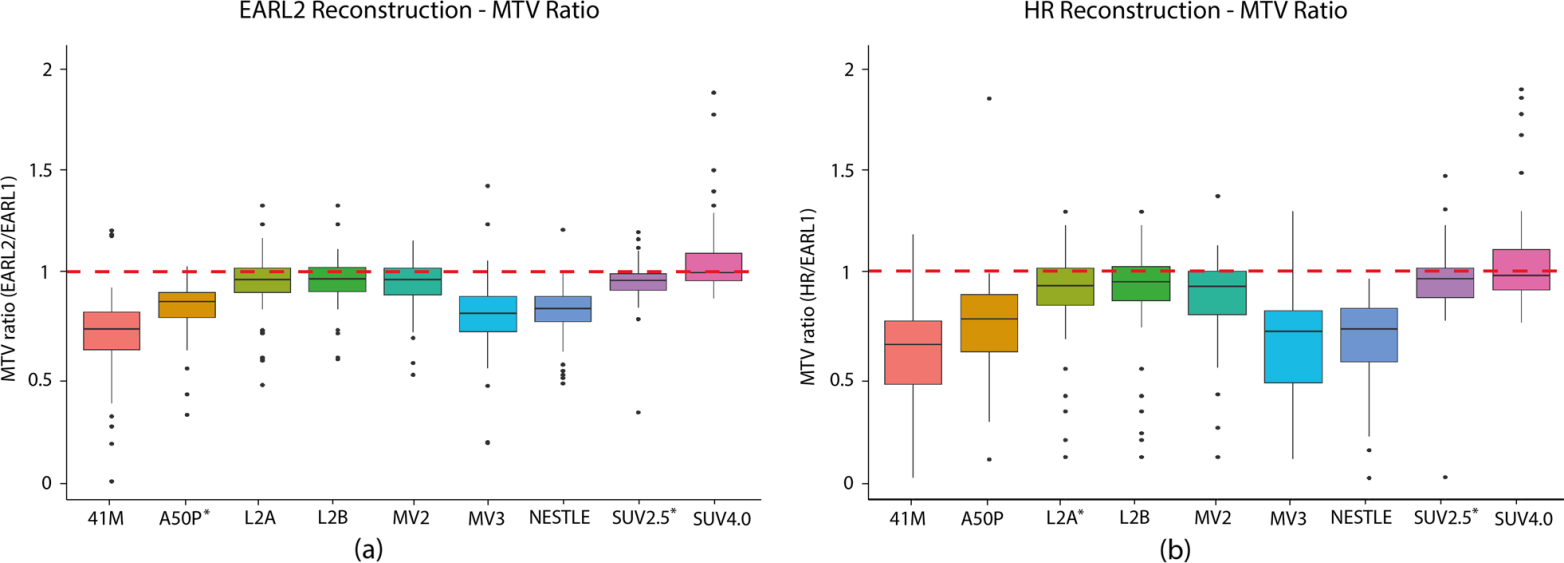
MTV ratios across segmentation methods. Each boxplot illustrates the set of MTV ratios obtained with a particular segmentation method: 41M, A50P, L2A, L2B, MV2, MV3, NESTLE, SUV2.5 or SUV4.0. MTV ratios are given for **a** EARL2 reconstructions and **b** HR reconstructions. * Implies few outliers not displayed

The MTV analysis was carried out by calculating the MTV volume ratios (see Eqs. 3 and 4 for reference). In Fig. 1, the MTV ratios are plotted per segmentation method for both EARL2 (a) and HR reconstructions (b). A perfect alignment of MTV values between reconstructions is given by an MTV ratio of 1. Both plots show dissimilarities with EARL1 reconstruction; however, MTV from the HR reconstruction presents larger variability than MTV from EARL2. Differences between reconstructions are readily apparent for segmentation methods 41M, A50P, MV3 and Nestle, where the MTV ratio boxplots stay below 1 (MTV ratio EARL2: median of 0.73, 0.86, 0.80 and 0.82, respectively), indicating that the volume of the lesions segmented under these settings is smaller than the segmented volume with EARL1 reconstructions. These findings are also presented in Table 3, where we displayed the median and IQR values for MTV ratios for each of the segmentation methods. In addition, for SUV2.5, the size and amount of outliers outnumbered the rest of segmentation methods (Additional file 1: Fig. S1). MTVs from the SUV4.0 segmentation method showed the best alignment between reconstructions (MTV ratio EARL2: median = 1.01, interquartile range (IQR) = [0.96, 1.10]). Generally, fixed threshold methods were less sensitive to changes in reconstruction settings (MTV ratio EARL2: median of 0.96 for MV2, L2A and L2B).

**Table 3:** MTV ratios (median and IQR for each segmentation method) for EARL2 and HR reconstructions

Segmentation	EARL2/EARL1			HR/EARL1		
	Median	IQR1	IQR3	Median	IQR1	IQR3
41M	0.73	0.63	0.81	0.65	0.45	0.76
A50P	0.86	0.79	0.90	0.77	0.61	0.89
L2A	0.96	0.90	1.02	0.93	0.84	1.03
L2B	0.96	0.91	1.02	0.95	0.86	1.02
MV2	0.96	0.89	1.02	0.92	0.79	1.00
MV3	0.80	0.72	0.88	0.71	0.46	0.81
NESTLE	0.82	0.76	0.88	0.72	0.56	0.82
SUV2.5	0.96	0.92	1.00	0.98	0.88	1.07
SUV4.0	1.01	0.96	1.10	1.00	0.91	1.16

*IQR* Interquartile Range

**Fig. 2:**
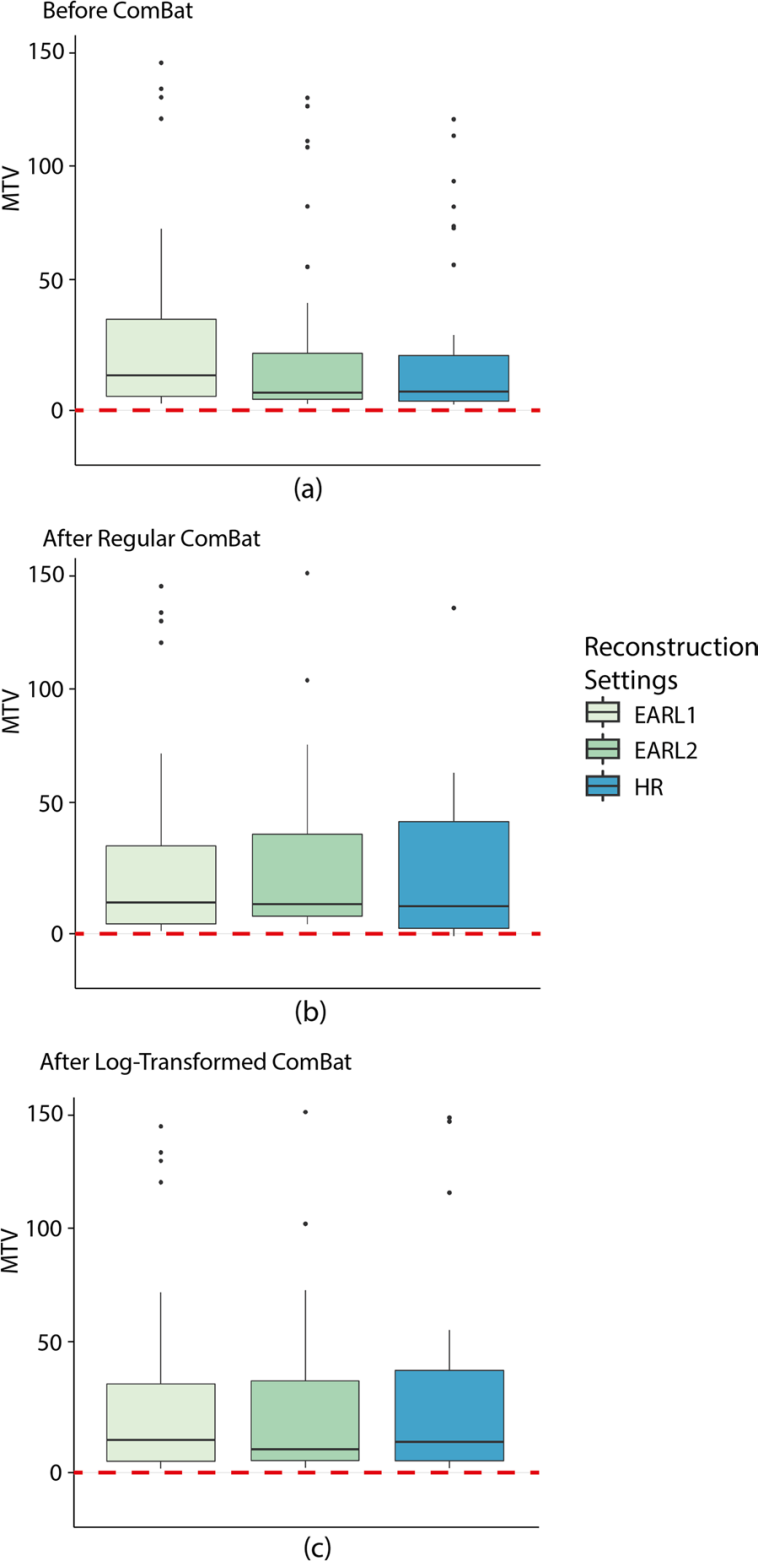
MTVs obtained using 41M segmentation across 3 different reconstructions: EARL1, EARL2 and HR. **a** MTVs before ComBat harmonization. **b** MTVs after ComBat using non-transformed distribution (Regular ComBat). **c** MTVs after ComBat using log-transformed distribution (Log-transformed ComBat). Regular ComBat leads to negative volume values for the clinical reconstruction unlike Log-transformed ComBat which led to positive-only volumes

ComBat transformation was applied to compensate for differences in reconstruction methods. Different versions of ComBat were implemented. In this paper, we focused on the comparison between the Regular ComBat and Log-transformed ComBat (see Table 2 for reference) because the latter is generally less sensitive to outliers and will, by definition, prevent the generation of negative values. Table 4 illustrates the median, mean, standard deviation (SD) and IQR of the MTVs before and after ComBat for 41M segmentation. SD and IQR are given because the data are not normally distributed. An improved alignment between reconstructions after using ComBat was observed. There is large variability in the data across the 3 reconstruction methods. This is also shown in Fig. 2. In Fig. 2, MTV values per reconstruction setting are presented using 41M segmentation method for all three situations: before ComBat (a), after Regular ComBat (b) and after Log-transformed ComBat (c). In Fig. 2b, negative MTVs were obtained for the HR reconstruction when using Regular ComBat. This was also observed for other segmentation methods such as SUV2.5, MV3 and A50P (Additional file 2: Fig. S2).

**Table 4:** Transformation of MTV values (mL) with ComBat harmonization

Method	Before ComBat		After regular ComBat		After Log-transformed ComBat	
	Mean (SD)	Median (IQR)	Mean (SD)	Median (IQR)	Mean (SD)	Median (IQR)
EARL1	118 (345)	13 (32)	118 (345)	13 (32)	118 (345)	13 (32)
EARL2	65 (196)	5 (19)	118 (345)	12 (34)	125 (385)	9 (33)
HR	52 (148)	6 (19)	118 (345)	11 (44)	100 (284)	12 (38)

*SD* Standard Deviation, *IQR* Interquartile Range

**Fig. 3:**
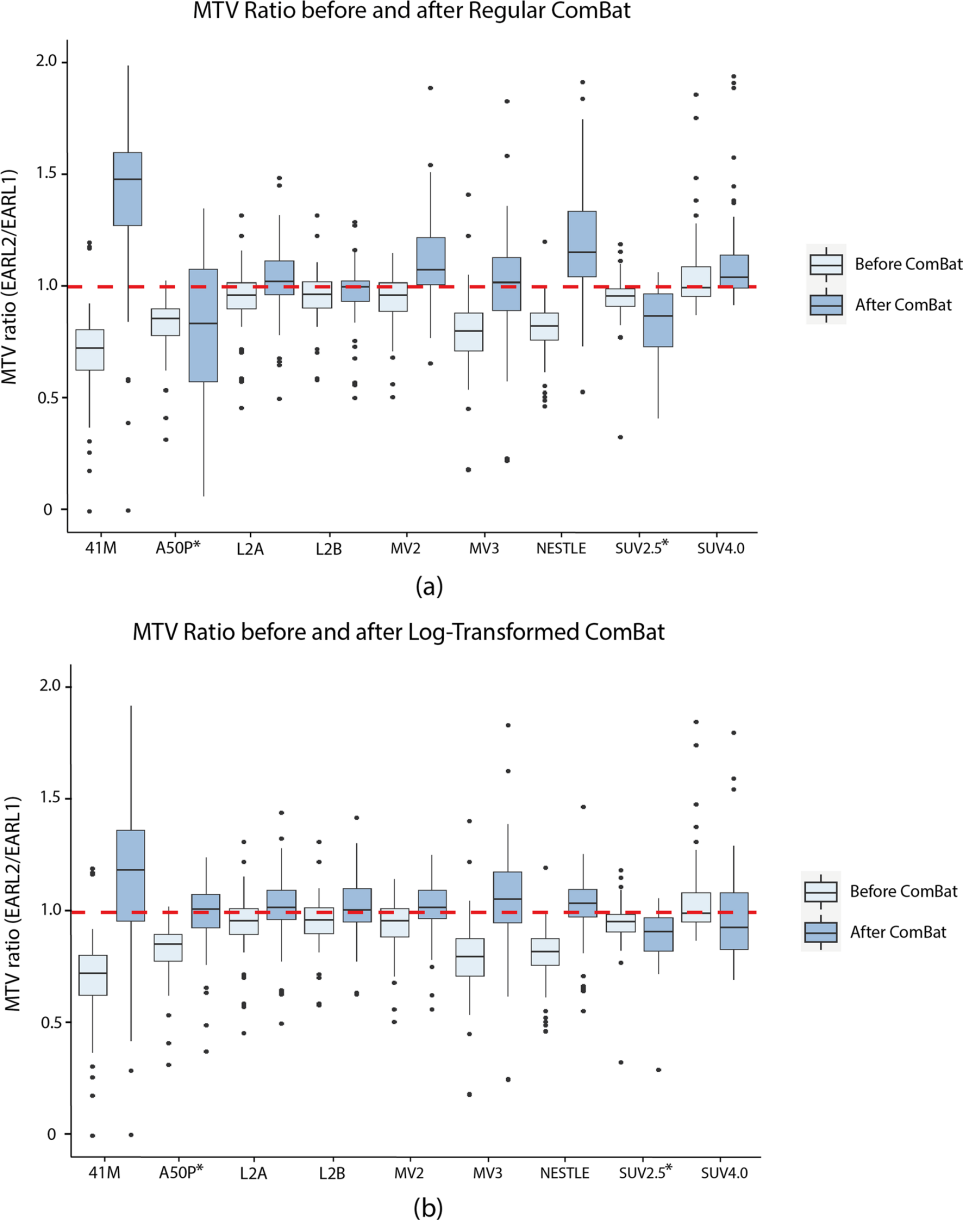
MTV ratio across 9 segmentation methods. MTV ratio calculated by comparing MTVs of EARL2 to those of EARL1, with EARL1 as the denominator for a particular segmentation method: 41M, A50P, L2A, L2B, MV2, MV3, NESTLE, SUV2.5 or SUV4.0. For each segmentation method, 2 boxplots are shown. The light blue boxplot represents data without ComBat harmonization, while the dark blue boxplot is obtained after ComBat harmonization. MTV ratio equal to 1 indicates a perfect alignment between reconstructions. **a** MTV ratio before and after ComBat using non-transformed distribution (Regular ComBat) **b** MTV ratio before and after ComBat using log-transformed distribution (Log-transformed ComBat)

The transformation of the MTV ratios per segmentation can be found in Fig. 3. Usually, an agreement of MTVs among different reconstructions can be observed post-ComBat harmonization for most segmentation methods. For most of the segmentation methods, the post-ComBat boxplots (dark blue) IQR included the value of 1, especially after applying the Log-transformed ComBat. However, these boxplots have larger IQR in comparison with the boxplots for the values before ComBat (light blue). This shows that ComBat increases the variability of the MTV parameter and consequently worsens the precision. The transformation was considerably better when applying Log-transformed ComBat instead of Regular ComBat. Log-transformed ComBat led to higher accuracy with median values closer to 1 and an acceptable increase in variability when compared to Regular ComBat.

## Discussion

The aim of this study was to evaluate the impact of image reconstruction methods onto MTV calculations in baseline [^18^F]FDG PET scans of lymphoma patients. For this study, we focused on two aspects: the reconstruction and the segmentation method. Specifically, we analyzed the interaction of three reconstruction methods with nine different segmentation methods and how these conditions affected MTV. At the moment, there is no consensus about which methods and settings are optimal for PET MTV quantification. However, the scientific community has acknowledged the need to generate robust and reproducible MTV measurements for prognostic and clinical applications [22–25].

The results of this study present significant inter-reconstruction variability for MTV calculations. The three different reconstruction methods which were evaluated (EARL1, EARL2 and HR) resulted, in some cases, in large differences in MTV values. Volumes derived from EARL2 and HR reconstructions have a tendency to be smaller in size when compared to EARL1. This is in concordance with a previous study where they found that PSF reconstructions led to a decrease in MTV in 83% of the analyzed lesions [26]. Furthermore, accurate MTV quantifications are also influenced by the segmentation method used. To our knowledge, this is the first study validating the effect of segmentation algorithms for different reconstruction methods, but the variation of MTV absolute values among segmentation methods has been previously reported in several studies [11, 25, 27]. Our results show that some segmentation methods are less sensitive to changes in reconstruction methods than others. The most robust (against reconstruction) segmentation method for MTV calculations was SUV4.0. In a recent work on DLBCL subjects, SUV4.0 was found to perform the best in deriving MTV compared to 6 other segmentation methods [19]. Results from MV2 segmentation were comparable to those of SUV4.0. MV2 segmentation tends to provide a fairly accurate segmentation of the lesions as it delineates voxels included in at least two out of four methods: SUV4.0, SUV2.5, A50P or 41% [11].

In the second stage of this study, we implemented ComBat with the aim of removing the variability introduced by the reconstructions. In a multicenter study on breast cancer [^18^F]FDG PET images, ComBat was successfully used to realign SUV measurements and multiple textural features [11]. Moreover, this approach has been validated for scanner effect removal in other imaging technologies such as CT [28] and MRI [29]. A better alignment in MTV between the different reconstruction methods was indeed accomplished once ComBat was applied. Our data showed very high values which caused large variability within reconstruction methods (Table 4). These extremely large values are generally originated by the presence of bulky tumors and the flooding effect caused by some segmentation methods. To deal with these extreme outliers, we used Log-transformed ComBat which achieves an improved alignment of MTV values between reconstructions compared to Regular ComBat. Some other versions of ComBat were implemented in the attempt to further remove this variability (using the median and the IQR in the transformation); however, these did not show a significant improvement. Despite a better alignment after ComBat, we still observed a decrease in accuracy and a worsened precision when comparing EARL2 and HR to EARL1. Therefore, an upfront harmonization of image quality and use of a consensus segmentation method are highly preferred. Furthermore, use of regular version of ComBat for the transformation resulted in negative MTV values for several segmentation methods particularly when using the HR reconstruction. Bearing this in mind, we believe ComBat should be used with caution. Adjusting the parameters of this method is important in order to avoid incoherent results and to mitigate any possible side effects of the ComBat harmonization.

The overall uncertainty and variability of PET extracted features can often be explained by the technical aspects involved in imaging acquisition. As such, unexpected deviations in volumetric features like MTV have to be carefully considered and should not be hastily adopted for response prediction. Novel technological implementations in reconstruction methods are significantly improving image quality standards; however, they have the effect of generating discrepancies in multicenter studies as not all PET systems can be equally equipped with these technologies. Lack of standardization is, therefore, becoming the main issue in MTV analysis of [^18^F]FDG PET-CT images. To address this matter, multiple medical societies like the European Association of Nuclear Medicine or the Society of Nuclear Medicine and Molecular Imaging are advocating for the inclusion of harmonized practices which can alleviate the variability and promote robust tumor quantification. Finally and most importantly, the harmonization of these methods is an essential step toward the implementation of MTV as a prognostic factor in clinical practice.

## Conclusion

This work corroborates the fact that robustness of MTV depends on both segmentation method and reconstruction methods. We found SUV4.0 to be the recommended method for lesion delineation, showing least sensitivity to image reconstruction settings. Moreover, ComBat was partially able to reduce reconstruction dependent MTV variability, provided a log-transformation to account for the non-normal distribution of MTVs is included. In conclusion, herein we demonstrate the impact of the imaging technical aspects in PET derived MTV and we highlight the importance of standardization in imaging workflows in order to enhance reproducibility for multicenter studies and, ultimately, the implementation of MTV for prognosis in clinical practice.

## Supplementary Information

**Additional file 1: Fig S1. **MTV Ratio values across segmentation methods including outliers. Each boxplot illustrates the set of MTV ratio values obtained with a particular segmentation method: 41M, A50P, L2A, L2B, MV2, MV3, NESTLE, SUV2.5 or SUV4.0. In **a** MTV ratios are given for EARL2 reconstruction and in **b** for HR reconstruction. SUV2.5 is the segmentation method with the greatest number of outliers and also with the highest values.

**Additional file 2: Fig S2**. MTVs after ComBat obtained using different segmentations across reconstructions. **a** Results obtained from MV3 segmentation. HR reconstruction shows negative MTVs. **b** Results obtained from A50P segmentation. The EARL2 reconstruction shows negative MTVs. **c** Results obtained from SUV2.5 segmentation. Both HR and EARL2 reconstructions show negative MTVs

## Data Availability

The datasets generated and/or analyzed during the current study are available from the corresponding author on reasonable request.

## Abbreviations

DLBCL: Diffuse large B cell lymphoma
PET: Positron emission tomography
CT: Computerized tomography
MTV: Metabolic tumor volume
[^18^F]FDG: [^18^F]-fluorodeoxyglucose
PFS: Progression-free survival
PSF: Point spread function
SUV: Standardized uptake value
PTLD: Post-transplant lymphoproliferative disorder
QC: Quality control
IQR: Interquartile range
