# Supplementary figures and images for: Combatting the effect of image reconstruction settings on lymphoma [18F]FDG PET metabolic tumor volume assessment using various segmentation methods
Source: EJNMMI Res. 2022 Jul 29;12:44. doi: 10.1186/s13550-022-00916-9 (PMC9338209; doi:10.1186/s13550-022-00916-9)

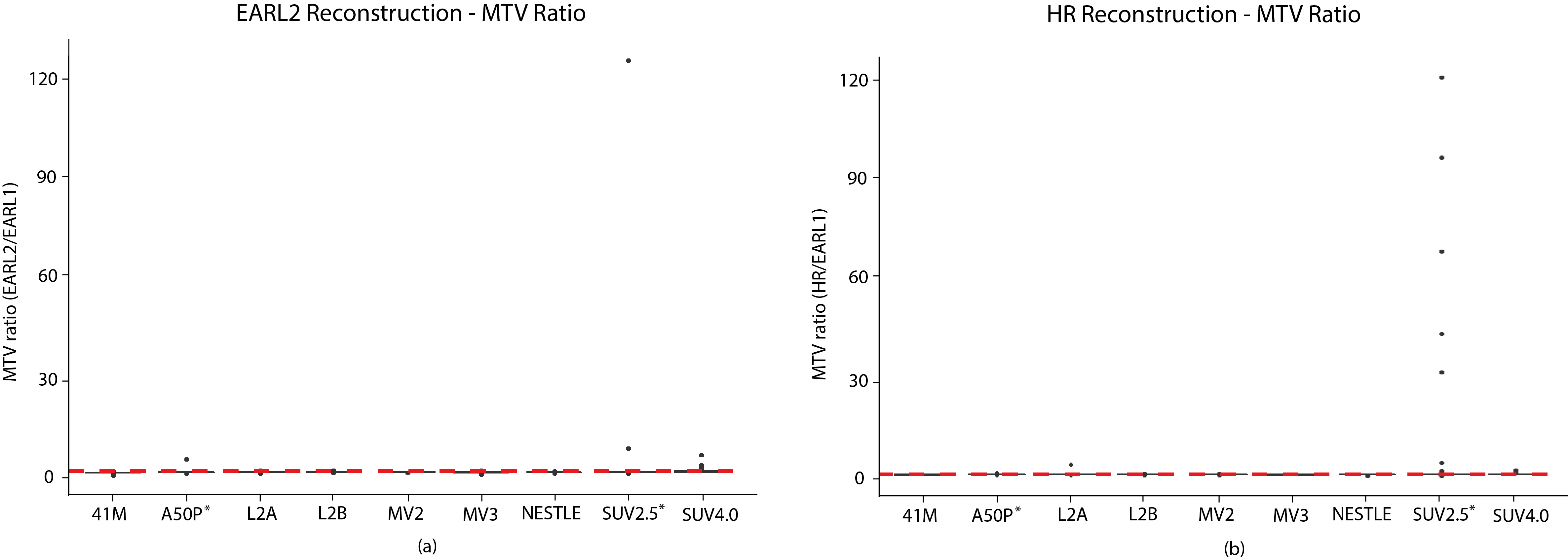

Supplement: Supplementary file 1 — Additional file 1: Fig S1. MTV Ratio values across segmentation methods including outliers. Each boxplot illustrates the set of MTV ratio values obtained with a particular segmentation method: 41M, A50P, L2A, L2B, MV2, MV3, NESTLE, SUV2.5 or SUV4.0. In a MTV ratios are given for EARL2 reconstruction and in b for HR reconstruction. SUV2.5 is the segmentation method with the greatest number of outliers and also with the highest values. [file 13550_2022_916_MOESM1_ESM.png]

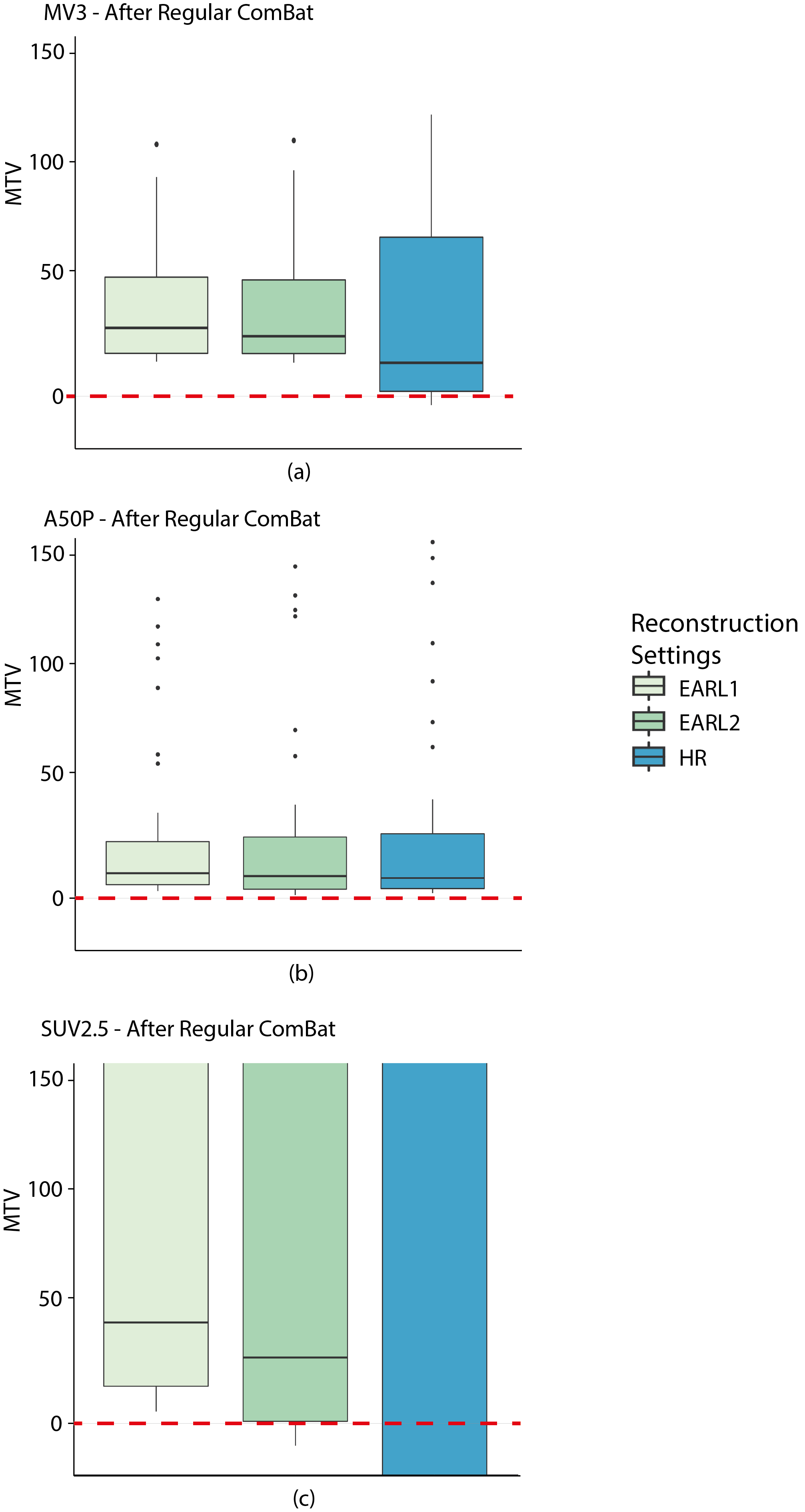

Supplement: Supplementary file 2 — Additional file 2: Fig S2. MTVs after ComBat obtained using different segmentations across reconstructions. a Results obtained from MV3 segmentation. HR reconstruction shows negative MTVs. b Results obtained from A50P segmentation. The EARL2 reconstruction shows negative MTVs. c Results obtained from SUV2.5 segmentation. Both HR and EARL2 reconstructions show negative MTVs [file 13550_2022_916_MOESM2_ESM.png]
